# Dynamic monitor on psychological problems of medical aid teams in the context of corona virus disease 2019: a multi-stage and multi-factor quantitative study

**DOI:** 10.1186/s12889-021-11479-0

**Published:** 2021-08-03

**Authors:** Yanji Zhou, Chengyu Li, Xiyu Zhang, Li Xu, Yuze Li, Wenqing Miao, Yingbo Dai, Dingyun You, Ye Li

**Affiliations:** 1 School of Public Health, Kunming Medical University, Kunming, 650500 Yunnan China; 2 Research Center of Public Policy and Management, School of Health Management, Harbin Medical University, Harbin, 150086 Heilongjiang China; 3 Department of Psychiatry, The Second Affiliated Hospital of Kunming Medical University, Kunming, 650101 Yunnan China; 4 Department of Medicine, Jiamusi University, Jiamusi, 154007 Heilongjiang China; 5 The First Affiliated Hospital of Harbin Medical University, Harbin, 150086 Heilongjiang China

**Keywords:** Mental health, Anxiety, Depression, Medical staff, COVID-19, China

## Abstract

**Background:**

To explore the psychological status and vulnerability characteristics of medical staff with the progress of the epidemic.

**Methods:**

This study investigated the prevalence of mental problems of 2748 medical staff in four stages. The PHQ-9 (Patient Health Questionnaire), GAD-7 (Generalized Anxiety Disorder questionnaire), SSS (Somatization Symptom Checklist), Pittsburgh sleep quality index, and PCL-C (Self-rating scale for post-traumatic stress disorder) were used for the psychological evaluation, and univariate logistic standardised analysis, and multivariate logistic regression for data analysis.

**Results:**

The prevalence of mental problems showed a statistically significant difference. In Stage 1, mild anxiety and mild depression reached the highest value of 41.4 and 40.72% respectively. Between 4 and 17 March that of mild depression rose from 16.07 to 26.7%, and between 17 and 26 March the prevalence of mild anxiety increased from 17.28 to 20.02%. Female, unmarried, and working in Wuhan are the risk factors of mental health of medical staff (*P* < 0.05).

**Conclusion:**

The psychological status of the medical staff has changed dynamically. Stage 1 and the latter period of Stages 2 and 3 are the high-risk stages. Female and unmarried are the dangerous characteristics of psychological vulnerability.

**Supplementary Information:**

The online version contains supplementary material available at 10.1186/s12889-021-11479-0.

## Introduction

At the end of 2019, no one expected that a potentially new malignant infectious disease was spreading quietly. Wuhan City became the target of the outbreak and was shrouded in panic by the unknown viral pneumonia. The Chinese government quickly launched a first-level emergency response plan and issued warning signals to the world. On 11 February 2020, the World Health Organization named this pandemic the Coronavirus Disease 2019 (COVID-2019), declaring a new round of confrontation between humans and infectious disease on a global scale. The cumulative number of confirmed cases increased from 291 on 21 January to 24,325 on 4 February. Around 15 February, China’s prevention and control of the epidemic began to achieve results. The number of new confirmed cases in China’s epidemic declined dramatically, and achieved zero cases reported for the first time on 19 March [1].

This health emergency situation tested Wuhan’s medical health system greatly, and nearly 42,000 medical staff all over the country were successively sent to Wuhan with medical resources [2]. Recent reports indicated that a European ICU (Intensive Care Unit) nurse committed suicide owing to excessive pressure under COVID-19. At that time, medical staff aiding Hubei Province also faced a large number of feverish and critically ill patients every day, and were under great physical and mental pressure [3]. Medical staff must not only be engaged in occupational pressure [4] and psychological counselling of patients with COVID-19 but also deal with the pressure owing to a shortage of materials, manpower, and unknown areas of the disease and epidemic [5].

Studies have confirmed that medical staff are more vulnerable to suffer psychological problems during the public health emergency period [6]. Negative psychological symptoms such as anxiety and depression were more likely to occur to medical staff, which was twice as likely to show anxiety or depression as the non-clinical staff [7]. Among them, the front-line medical staff were more likely to suffer from anxiety and depression, thus, suffering from the impact of mental illness [8]. In addition, occupation [9], marital status [10], and education level [11] also proved to be fragile characteristics of medical staff suffering from psychological problems during COVID-19. However, to our knowledge, almost all of this literature stayed in the cross-sectional study, and research on multi-periods comparison of the psychological status of first-line medical staff is still a relative blank stage. Dynamic monitoring of the psychological status of the medical staff is significant to target the characteristics of vulnerable groups and further make precise intervention plans in the catastrophic health emergencies period, which is crucial not only for the work performance of the medical staff but also for the quality care of patients [12]. Accordingly, our study is to evaluate the psychological status of medical staff for four key time points in the COVID-19 epidemic, and to make up for the blank in current research on the dynamic monitoring of the psychological status of medical staff innovatively. By multi-dimensional assessment, the intervention priorities of vulnerable groups will be screened accurately, which provides good experience for other countries still in the pandemic storm.

## Methods

### Design and sample procedure

**Fig. 1:**
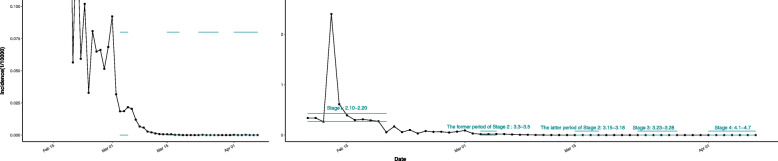
The incidence trend of COVID-19 and the time points of this study

There are 60 questions in the questionnaire (including primary demographic questions, the Pittsburgh sleep post-traumatic stress disorder (PCL-C), GAD-7 anxiety screening scale, the PHQ-9 depression screening scale, the Somatization Symptom Checklist (SSS), etc.). The depression level was assessed by the PHQ-9 depression screening scale, the anxiety level was assessed by the GAD-7 anxiety screening scale, and physical disorder was assessed by the Somatization Symptom Checklist (SSS), the Pittsburgh sleep post-traumatic stress disorder (PCL-C) was used for the evaluation of post-traumatic stress disorder. (The grouping is shown in Appendix Table 1. Due to the small number of samples in some groups, for analysis, we combined the “moderate”, “severe” and “very severe” groups.) And all the data were collected via an online survey agency (Wenjuanxing www.wjx.cn). However, considering that the topic of this paper is the comparison of depression and anxiety between doctors and non-doctors, this study only used 27 questions about anxiety (10 questions), depression (9 questions) and basic demographic information (8 questions) which is highly relevant to this study. And other items of the questionnaire (Pittsburgh sleep quality index, Somatization Symptom Checklist, trauma stress rating scale) are not used, so it is considered to be put in another paper with a more appropriate topic. The subjects of this study are all the medical staff aiding Hubei Province from Yunnan Province, although a few of them did not respond to the survey. A total of 2748 medical staff (from medical teams aiding Hubei Province of hospitals in Yunnan Province, including rheumatology and immunology department, rehabilitation medicine department, respiratory and critical care department, endocrinology department and so forth) were investigated at four stages (five-time points): Stage 1 (10 to 20 February: the beginning of the epidemic), Stage 2 (3 to 5 March, 15 to 18 March: the peak of the epidemic), Stage 3 (23 to 28 March: returning time from Hubei Province), and Stage 4 (1 to 7 April: time after isolation and recuperation) with a response rate of 39.18, 31.81, 68.71, 93.88, and 88.65% respectively. Figure 1 shows the incidence trend during the epidemic period and the investigation time points of this study.

### Statistical analysis

In this study, the statistical analysis was implemented by R 3.6.2 software, and the graphs were drawn with the R 3.6.2 software ggplot2 package. After the data cleaning, a total of 2748 medical staff who participated in the questionnaire at five time points were included as the total number of observation cases. According to the depression and anxiety scale, the symptoms of all participants were divided into three levels (none, mild, moderate, and severe). Based on the literature review, determinants of depression and anxiety included: gender, occupation, age, marital status, educational background, workplace during the epidemic. Logistic regression analysis, univariate logistic stratified analysis (time stratification), and multivariate logistic regression analysis were used to analyse the correlation between depression (anxiety) level. We established the significance of the two-sided test as *P* < 0.05.

In a univariate logistic stratified analysis, time was used as a stratified variable. For the variable ‘working place during the epidemic’, the option of ‘working in regions outside Hubei Province’ could not be stratified because of too many missing values. In contrast, options of ‘working in Wuhan’ and ‘working in regions in Hubei Province except for Wuhan’ were all based on the medical aid team’s isolation and recuperation time (Stage 4) as the reference level. For the variable ‘occupation’, ‘other’ was based on when the medical team returned to the Yunnan Province (Stage 3); the other options were based on the reference level at the beginning of the epidemic (Stage 1).

To explore the relationship between the different time nodes of the epidemic development and degree of depression and anxiety of medical staff, the multi-factor logistic regression analysis was used. A total of 2748 medical staff were selected as the observation objects, and the independent variables were different time nodes (10 to 20 February, 3 to 5 March, 15 to 18 March, 23 to 28 March, 1 to 7 April), depression level (no depression, mild depression, moderate, and severe depression) and anxiety (no anxiety, mild anxiety, moderate, and severe anxiety) of medical staff. Taking Stage 1 (10 to 20 February) as the reference level, the OR value (95% CI) and *P* value were observed during Stage 2 (3 to 5 March, 15 to 18 March), Stage 3 (23 to 28 March), and Stage 4 (1 to 7 April). Model 1 was a single factor logistic regression model without adjusting any variables; Model 2 adjusted some variables (gender, age, and occupation of medical staff); Model 3 adjusted all variables (gender, age, occupation, marital status, and education level) to control the bias caused by confounding factors.

## Results

### Basic information

A total of 2748 participants were investigated in all the four stages. In Stage 1, 180 (40.82%) doctors and 261 (59.18%) nurses were investigated. In Stage 3, 264 (24.93%) doctors and 773 (73.0%) nurses were investigated. In Stage 4, 254 (25.43%) doctors and 721 (72.17%) nurses participated in the survey. A total of 442 medical staff working outside Hubei Province were investigated at Stage 1 (the beginning of the epidemic), which was the main period of investigation for medical staff working outside Hubei Province. From Stage 3 on, medical staff working in Hubei Province were investigated. In Stage 3, 601 (57.46%) medical workers were in Wuhan and 444 (42.45%) medical workers were in other cities of Hubei Province. Importantly, the female medical staff were larger than the male staff, especially in Stage 1, which investigated 362 (81.90%) women and 80 (18.10%) men. In addition, the population we investigated consisted mainly of medical workers aged 21 to 40. In Stage 3, 382 (36.17%) medical workers in their 20s and 432 (40.91%) medical workers in their 30s were investigated. This was the most significant stage (Table 1).

**Table 1:** Table of research object composition

Variables	Date									
	Stage 1 (10.Feb-20.Feb)		Stage 2 (3.Mar-5.Mar)		Stage 2 (15.Mar-18.Mar)		Stage 3 (23.Mar-28.Mar)		Stage 4 (1.Apr-7.Apr)	
**Work Location**										
**Wuhan**							601	(57.46%)	577	(58.34%)
**Regions outside Hubei Province**	442	(100.00%)					1	(0.10%)	1	(0.10%)
**Regions of Hubei Province except Wuhan**							444	(42.45%)	411	(41.56%)
**Sex**										
**Male**	80	(18.10%)	16	(28.57%)	70	(36.65%)	298	(28.14%)	294	(29.40%)
**Female**	362	(81.90%)	40	(71.43%)	121	(63.35%)	761	(71.86%)	706	(70.60%)
**Occupation**										
**Doctor**	180	(40.82%)	18	(32.14%)	70	(36.65%)	264	(24.93%)	254	(25.43%)
**Nurse**	261	(59.18%)	38	(67.86%)	120	(62.83%)	773	(72.99%)	721	(72.17%)
**Other**					1	(0.52%)	22	(2.08%)	24	(2.40%)
**Age**										
**21–30**	185	(41.86%)	20	(35.71%)	87	(45.55%)	382	(36.17%)	352	(35.24%)
**31–40**	149	(33.71%)	15	(26.79%)	67	(35.08%)	432	(40.91%)	410	(41.04%)
**41–50**	83	(18.78%)	20	(35.71%)	35	(18.32%)	218	(20.64%)	209	(20.92%)
**51–60**	20	(4.52%)			2	(1.05%)	23	(2.18%)	28	(2.80%)
**> 60**	5	(1.13%)	1	(1.79%)			1	(0.09%)		
**Marital Status**										
**Unmarried**	115	(26.02%)	14	(25.00%)	65	(34.03%)	289	(27.29%)	266	(26.60%)
**Married**	317	(71.72%)	39	(69.64%)	122	(63.87%)	734	(69.31%)	700	(70.00%)
**Other**	10	(2.26%)	3	(5.36%)	4	(2.09%)	36	(3.40%)	34	(3.40%)
**Educational Background**										
**Undergraduate**	292	(66.06%)	46	(82.14%)	140	(73.30%)	772	(72.90%)	730	(73.00%)
**Graduate**	67	(15.16%)	1	(1.79%)	14	(7.33%)	103	(9.73%)	87	(8.70%)
**Middle School**	83	(18.78%)	9	(16.07%)	37	(19.37%)	184	(17.37%)	183	(18.30%)

### Anxiety

According to our survey data, a statistical correlation between anxiety level and the occurrence and development of epidemic diseases (χ^2^ = 206.394, *P* < 0.0001) showed. In Stage 1, the prevalence of moderate and severe anxiety was 7.47% and kept decreasing, which gradually decreased to 2.46% in Stage 3 and even to 0.9% in Stage 4. The prevalence of mild anxiety was 41.4% in Stage 1, with the downward trend to 17.28% in Stage 2, and finally to 12.4% in Stage 4. In all subgroups, most of the respondents were those without anxiety symptoms, and the proportion of respondents with moderate and severe anxiety was the smallest. Further stratified analysis showed that various factors had a certain impact on the anxiety variables of medical staff. Compared with working in Wuhan City, working in regions outside Wuhan especially outside Hubei Province was more likely to have a higher level of anxiety (OR,4.566;95%CI,3.603–5.786). Univariate logistic regression analysis showed that gender was associated with anxiety (male medical staff vs female medical staff (OR,0.639;95% CI,0.517–0.79). Further, compared with 21–30-year-old young medical staff, those over 40 years old were inclined to a greater risk of anxiety (OR,1.464;95%CI,1.156–1.856). Married (OR,1.982;95%CI,1.047,3.750) and unmarried (OR,2.394;95%CI,1.250,4.585) medical workers were more probable to suffer from anxiety problems than medical workers with other marital status. In addition, compared to the undergraduate education group, the graduates’ educational background group was more likely to suffer anxiety. (OR,1.330;95CI,1.002,1.767) **(**Table 2**)**.

**Table 2:** Factors influencing anxiety level

Variables		No anxiety (***N*** = 1924)	Mild anxiety (***N*** = 656)	Moderate and Severe anxiety (***N*** = 168)	χ^2^	P	OR(95%CI)
Time					206.3935	<.0001	
10-Feb ~ 20-Feb	442	226(51.13)	183(41.4)	33(7.47)	/	/	1
3-Mar ~ 5-Mar	56	44(78.57)	12(21.43)	0(0)	0.2995	0.5842	0.276(0.142–0.537)
15-Mar ~ 18-Mar	191	154(80.63)	33(17.28)	4(2.09)	2.2291	0.1354	0.25(0.167–0.374)
23-Mar ~ 28-Mar	1059	821(77.53)	212(20.02)	26(2.46)	0.3158	0.5741	0.302(0.239–0.381)
1-Apr ~ 7-Apr	1000	867(86.7)	124(12.4)	9(0.9)	41.4921	<.0001	0.159(0.123–0.206)
**Work Location**					181.0609	<.0001	
Wuhan	1178	974(82.68)	186(15.79)	18(1.53)	/	/	1
Regions outside Hubei Province	444	228(51.35)	183(41.22)	33(7.43)	177.7324	<.0001	4.566(3.603–5.786)
Regions of Hubei Province except Wuhan	855	693(81.05)	147(17.19)	15(1.75)	37.4884	<.0001	1.117(0.889–1.402)
**Sex**					17.1784	<.0001	
Male	758	624(82.32)	118(15.57)	16(2.11)	17.1784	<.0001	0.639(0.517–0.79)
Female	1990	1488(74.77)	446(22.41)	56(2.81)	/	/	1
**Occupation**					0.8661	0.6485	
Doctor	786	614(78.12)	148(18.83)	24(3.05)	/	/	1
Nurse	1913	1459(76.27)	409(21.38)	45(2.35)	0.2209	0.6384	1.097(0.9–1.338)
Other	47	37(78.72)	7(14.89)	3(6.38)	0.0114	0.9151	1.009(0.498–2.044)
**Age**					10.7989	0.001	
21–30	1026	750(27.33)	249(9.07)	27(0.98)	/	/	1
31–40	1073	843(30.72)	199(7.25)	31(1.13)	0.9832	0.3214	1.33(1.089–1.625)
> 40	645	516(18.8)	116(4.23)	13(0.47)	4.6522	0.031	1.464(1.156–1.856)
**Marital Status**					8.8586	0.0119	
Unmarried	749	553(73.83)	179(23.9)	17(2.27)	8.455	0.0036	2.394(1.250,4.585)
Married	1912	1483(77.56)	376(19.67)	53(2.77)	2.0094	0.1563	1.982(1.047,3.750)
Other	87	76(87.36)	9(10.34)	2(2.3)	/	/	1
**Educational Background**					3.8925	0.1428	
Undergraduate	1980	1534(77.47)	396(20)	50(2.53)	/	/	1
Graduate	272	197(72.43)	63(23.16)	12(4.41)	3.3847	0.0658	1.330(1.002,1.767)
Middle School	496	381(76.81)	105(21.17)	10(2.02)	0.7725	0.3795	1.030(0.816,1.301)

*10.Feb-20.Feb is at the beginning of coronavirus epidemic in China,

3.Mar-5.Mar and 15.Mar-18.Mar is in the period of coronavirus epidemic in China

Medical support teams backed in 23.Mar-28.Mar.

1.Apr-7.Apr:after 14 days rest

**Fig. 2:**
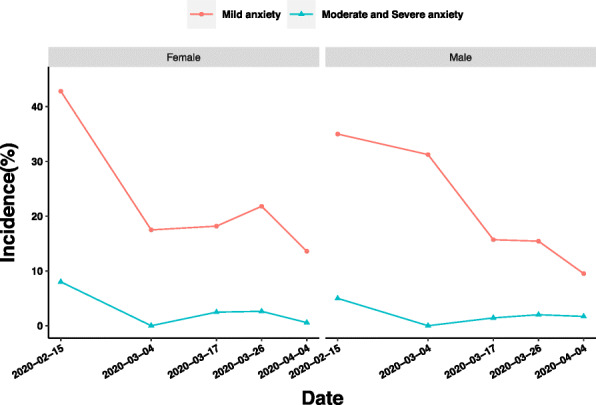
Influence of gender on anxiety of medical staff in different periods of epidemic

Obviously, compared with the medical staff who felt mild anxiety, the medical staff who felt moderate or severe anxiety constituted a small part of the respondents. As shown in Fig. 2, the prevalence of mild anxiety in males have been decreasing all the time, but the prevalence of mild anxiety in women has increased slightly from 17.5% (the former period of Stage 2) to 21.81% (Stage 3) (4 March to 26 March). In Stage 1, both the male and female medical staff were found to suffer the highest prevalence of mild anxiety with 42.82% for female medical staff, which was 7.82% higher than that of male staff. Moreover, the prevalence of moderate and severe anxiety of medical workers of different genders were highest to 5% for male medical workers and 8.01% for female medical workers, then decreased to the lowest prevalence in the former period of Stage 2. Although this prevalence slightly increased to 2.63% for female and 2.01% for male, it decreased to 0.57 and 1.7% for female and for male, respectively in Stage 4.

**Fig. 3:**
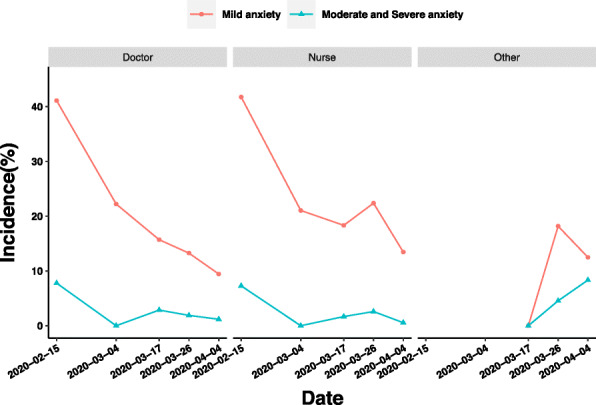
Influence of occupation on anxiety of medical staff in different periods of epidemic

For nurses, the prevalence of mild anxiety increased from 18.33% (the latter period of Stage 2) to 22.38% (Stage 3), although the changing trends of anxiety of doctors and nurses are similarly downward. Moreover, the main difference of moderate and severe anxiety is that the prevalence of doctors decreased from 2.86% (the latter period of Stage 2) to 1.89% (Stage 3), while that of nurses increased from 1.67% (the latter period of Stage 2) to 2.59% (Stage 3) (Fig. 3).

**Fig. 4:**
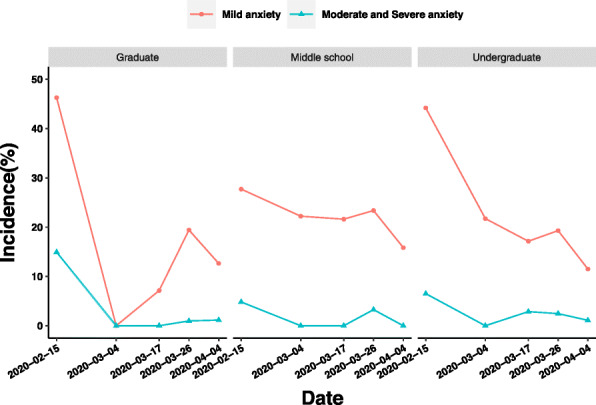
Influence of educational background on anxiety of medical staff in different periods of epidemic

In Fig. 4, from the first sampling (Stage 1) to 17 March (the latter period of Stage 2), the prevalence of mild anxiety in graduate education background group, undergraduate education background group, and middle school education background group decreased by 39.13, 27.04, and 6.09%, respectively. The main difference of the prevalence of moderate and severe anxiety of medical staff with different educational backgrounds occurred between Stages 3 and 4. The prevalence of moderate and severe anxiety of medical staff with graduate education increased from 0.97 to 1.15% between Stages 3 and 4, while the prevalence of moderate and severe anxiety of medical staff with undergraduate education background decreased from 2.46 to 1.1% during the same period.

**Fig. 5:**
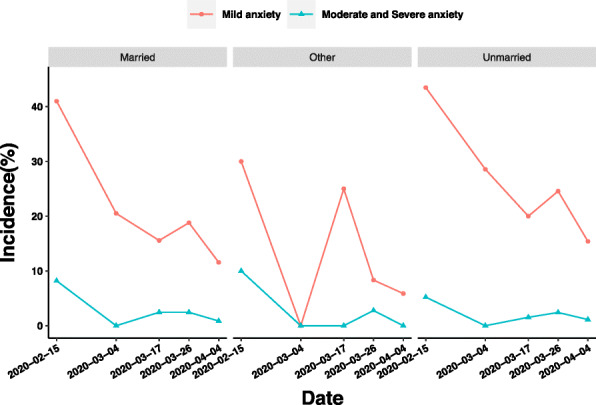
Influence of marriage on anxiety of medical staff in different periods of epidemic

Although the prevalence of mild anxiety of married and unmarried medical staff increased by 3.23 and 4.57%, respectively, only between the latter period of Stages 2 and 3, while the prevalence of medical staff with other marital status decreased sharply from 25 to 8.33%. The prevalence of moderate and severe anxiety of medical staff in different marital status groups was similar, but one point that required attention was between the latter period of Stages 2 and 3, the prevalence of moderate and severe anxiety of married medical staff decreased by 0.01%, while that of unmarried medical staff increased by 0.88% (Fig. 5).

### Depression

Our survey data, showed a statistical relationship between the level of depression and the occurrence and development of the pandemic (χ^2^ = 181.739, *P* < 0.0001). The prevalence of moderate and severe anxiety in Stage 1 was 14.48%, which continued to decline to 2.09% in Stage 2 and increased to 6.61% in Stage 3, but finally declined to 3% in Stage 4. The highest prevalence of mild anxiety of depression was 40.72% in Stage 1, and then decreased to 0.39 times of the original in the former period of Stage 2. However, from the latter period of Stage 2 onwards, it temporarily rose to 26.7% in the latter period of Stage 2, then continued to decline to 17.4% in Stage 4.

In all subgroups, the proportion of moderate and severe depression was less than that of mild depression. A further stratified analysis showed that all kinds of variables of interest also had a certain impact on the depression variables of medical staff. Compared with working in Wuhan, working outside Wuhan, especially in regions outside Hubei Province, is more likely to reach higher depression levels (OR,3.846;95%CI,3.079–4.804). Regression analysis showed that gender was associated with depression (male versus female; OR,0.676;95%CI, 0.581–0.786). Moreover, the risk of depression was higher in the medical staff over 40 years old than in the young medical staff aged 21–30 years (OR,1.577;95%CI,1.268–1.961). Unmarried medical staff were more at risk of depression than married medical staff (OR,1.325;95%CI, 1.109–1.583), while those with other marital status were less at risk of depression than married medical staff (OR,0.858;95%CI,0.526–1.399). In addition, compared with the undergraduate education background group, the graduate education background group is more likely to produce a higher prevalence of anxiety (OR,1.120;95CI,0.857–1.465) (Table 3).

**Table 3:** Factors influencing depression level

Variables		No Depression (*N* = 1924)	Mild Depression(*N* = 656)	Moderate and Severe Depression (*N* = 168)	χ^2^	P	OR(95%CI)
Time					181.7389	<.0001	
10-Feb ~ 20-Feb	442	198(44.8)	180(40.72)	64(14.48)	/	/	1
3-Mar ~ 5-Mar	56	47(83.93)	9(16.07)	0(0)	6.4561	0.0111	0.151(0.072–0.317)
15-Mar ~ 18-Mar	191	136(71.2)	51(26.7)	4(2.09)	0.016	0.8993	0.315(0.219–0.451)
23-Mar ~ 28-Mar	1059	747(70.54)	242(22.85)	70(6.61)	0.5384	0.4631	0.345(0.277–0.43)
1-Apr ~ 7-Apr	1000	796(79.6)	174(17.4)	30(3)	17.6115	<.0001	0.207(0.164–0.262)
**Work Location**					153.0325	<.0001	
Wuhan	1178	896(76.06)	235(19.95)	47(3.99)	/	/	1
Regions outside Hubei Province	444	200(45.05)	180(40.54)	64(14.41)	148.1195	<.0001	3.846(3.079–4.804)
Regions of Hubei Province except Wuhan	855	628(73.45)	177(20.7)	50(5.85)	29.5819	<.0001	1.168(0.955–1.427)
**Sex**					30.6478	<.0001	
Male	758	592(78.1)	128(16.89)	38(5.01)	30.6478	<.0001	0.676(0.581–0.786)
Female	1990	1332(66.93)	528(26.53)	130(6.53)	/	/	1
**Occupation**					2.3018	0.3163	
Doctor	786	568(72.26)	169(21.5)	49(6.23)	/	/	1
Nurse	1913	1322(69.11)	475(24.83)	116(6.06)	0.0737	0.786	1.148(0.957–1.378)
Other	47	32(68.09)	12(25.53)	3(6.38)	0.1399	0.7084	1.205(0.644–2.252)
**Age**					20.9162	0.0003	
21–30	1026	673(24.53)	276(10.06)	77(2.81)	/	/	1
31–40	1073	764(27.84)	251(9.15)	58(2.11)	0.2071	0.649	1.307(1.089–1.568)
> 40	645	484(17.64)	129(4.7)	32(1.17)	10.0366	0.0015	1.577(1.268–1.961)
**Marital Status**					10.5116	0.0052	
Unmarried	749	492(65.69)	198(26.44)	59(7.88)	6.0112	0.0142	1.325(1.109,1.583)
Married	1912	1368(71.55)	436(22.8)	108(5.65)	/	/	1
Other	87	64(73.56)	22(25.29)	1(1.15)	1.3968	0.2373	0.858(0.526,1.399)
**Educational Background**					1.19	0.5516	
Undergraduate	1980	1388(70.1)	466(23.54)	126(6.36)	/	/	1
Graduate	272	184(67.65)	69(25.37)	19(6.99)	1.0826	0.2981	1.120(0.857,1.465)
Middle School	496	352(70.97)	121(24.4)	23(4.64)	0.988	0.3202	0.940(0.758,1.165)

*10.Feb-20.Feb is at the beginning of coronavirus epidemic in China,

3.Mar-5.Mar and 15.Mar-18.Mar is in the period of coronavirus epidemic in China

Medical support teams backed in 23.Mar-28.Mar.

1.Apr-7.Apr:after 14 days rest

**Fig. 6:**
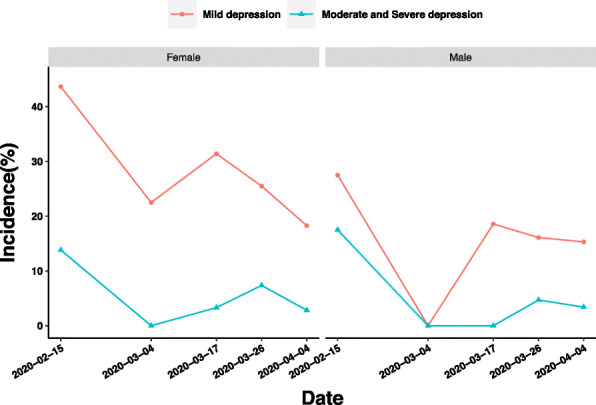
Influence of gender on depression of medical staff in different periods of epidemic

The prevalence of mild anxiety of female medical staff decreased by 5.91 and 7.22%, respectively, between the latter period of Stages 2 and 3, and between Stages 3 and 4, while that of male medical staff decreased by 2.46 and 0.8%, respectively. Between Stages 3 and 4, the prevalence of moderate and severe anxiety in male and female medical staff decreased by 1.3 and 4.53%, respectively, which indicated that women responded more intensely to the changing variables of interest in this stage (Fig. 6).

**Fig. 7:**
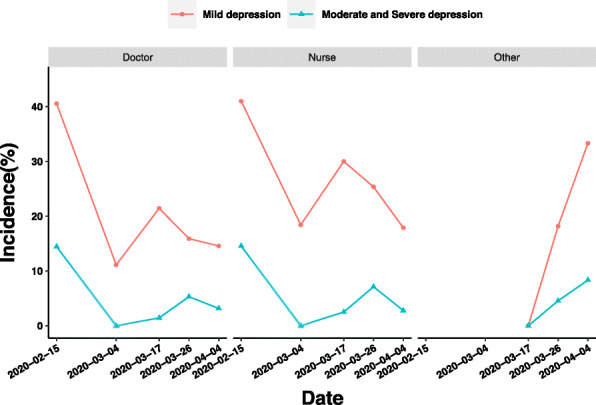
Influence of occupation on depression of medical staff in different periods of epidemic

We found that nurses’ prevalence of mild depression and moderate to severe depression decreased by 7.47 and 4.35% between Stages 3 and 4, respectively. In contrast, doctors’ prevalence of mild depression and moderate to severe depression decreased by 1.34 and 2.15% between Stages 3 and 4, respectively, which shows that the reaction of nurses to the change factors between Stages 3 and 4 is more intense (Fig. 7).

**Fig. 8:**
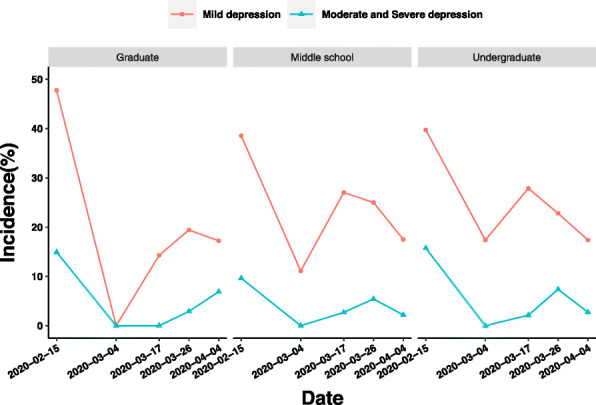
Influence of educational background on depression o medical staff in different periods of epidemic

The mild depression of medical staff with different educational backgrounds showed a great difference between the latter period of Stages 2 and 3. During this period, graduate students’ prevalence of mild depression increased from 14.29 to 19.42%. However, the prevalence of mild depression decreased by 5.06% in undergraduate education background group and 2.03% in middle school education background group. The main difference of moderate and severe depression among medical staff with different educational backgrounds existed between Stages 3 and 4. During this period, the prevalence of moderate and severe depression of medical staff with undergraduate education and middle school education decreased, but the prevalence of moderate and severe depression of medical staff with graduate education increased by 3.99% (Fig. 8).

**Fig. 9:**
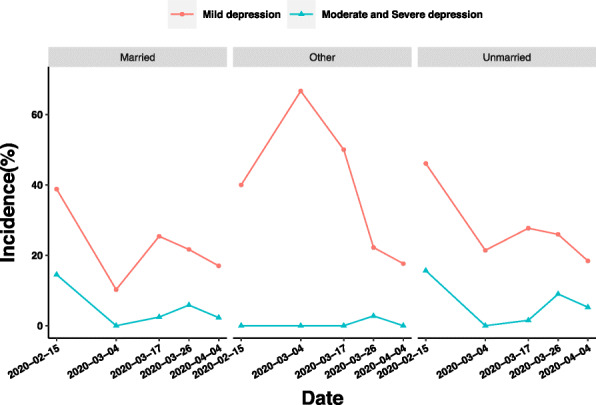
Influence ofs marriage on depression of medical staff in different periods of epidemic

Between Stage 1 and the former period of Stage 2 (3 to 5 March), only the prevalence of mild depression of medical staff with other marital status increased from 40 to 66.67%, and the prevalence of mild depression of married and unmarried medical staff was alleviated. However, the prevalence of mild depression in medical staff with other marital status has been declining since then, while the prevalence of mild depression in married and unmarried medical staff increased by 15.15 and 6.26%, respectively, between the two periods of Stage 2 (4 to 17 March 17). The difference of moderate and severe depression among medical staff with different marital status requires attention between the latter period of Stages 2 and 3. During this period, the prevalence of moderate and severe depression of married medical staff increased from 2.46 to 5.86%, while that of unmarried medical staff increased from 1.54 to 9% (Fig. 9).

### Analysis of the influencing factors of anxiety and depression

A total of 2748 medical staff were observed, applying the multivariate regression model to explore the relevance between time and depression/anxiety of staff. Variable ‘Time’ was taken as an independent variable, depression/anxiety level of medical staff were taken as dependent variables. Stage 1 (10 to 20 February) was taken as a reference in multiple logistic regression, and OR (95%CI) and *P* value were observed. Different models based on different adjustments were built to control the potential bias of the confounder. In Model 1, we did a single factor analysis, and any variables were adjusted. In Model 2, staff’s gender, age, and occupation were adjusted. In Model 3, staff’s gender, age, occupation, marital status, and educational background were adjusted.

In a logistic regression analysis of depression in staff, comparing with Stage 1 (10 to 20 February), the proportion of depression in staff in other times (3 to 5 March, 15 to 18 March, 23 to 28 March, 1 to 7 April) were all observed declined with different decreases. The decrease observed was statistically different in the former period of Stage 2 (3 to 5 March) and the decrease of Stage 4 (1 to 7 April: after medical support teams got rest) also observed a statistically significant difference. According to P for trend, the decline of the proportion of depression in staff as time goes by were observed as a statistically significant difference. The same trend was observed both in Models 2 and 3.

A similar trend was found in the results of the logistic regression analysis of anxiety in staff. Compared with Stage 1 (10 February to 20 February), the proportion of anxiety in staff in other times (3 to 5 March, 15 to 18 March, 23 to 28 March, 1 to 7 April) declined with different decreases. A decrease of Stage 4 (1 to 7 April: after medical support teams got rest) was observed as statistically significant different. According to P for trend, the decline of the proportion of anxiety in staff as time passed was a statistically significant difference. The same trend was observed both in Models 2 and 3 **(**Table 4**,** Table 5**)**.

**Table 4:** Multivariate analysis results of anxiety

Time	Model 1(*cRR*)	Model 2(*aRR*)	Model 3(*aRR*)
10-Feb ~ 20-Feb	Ref	Ref	Ref
3-Mar ~ 5-Mar	0.276(0.142,0.537)	0.279(0.143,0.545)	0.278(0.142,0.544)
15-Mar ~ 18-Mar	0.25(0.167,0.374)	0.251(0.167,0.376)	0.249(0.166,0.374)
23-Mar ~ 28-Mar	0.302(0.239,0.381)	0.293(0.23,0.371)	0.293(0.231,0.372)
1-Apr ~ 7-Apr	0.159(0.123,0.206)***	0.155(0.119,0.202)***	0.155(0.119,0.202)***
*P-trend*	0.655(0.617,0.696)***	0.650(0.611,0.692)***	0.650(0.611,0.692)***

Model 1: Single factor analysis. Model 2: Staff’s sex, age and occupation were adjusted. Model 3: Staff’s sex, age, occupation, marital status, and educational background were adjusted. cRR: cursory Risk Ratio. aRR: adjusted Risk Ratio.

*P < 0.05, **P < 0.01,***P < 0.0001。

*10-Feb ~ 20-Feb is at the beginning of coronavirus epidemic in China, 3-Mar ~ 5-Mar and 15-Mar ~ 18-Mar is in the period of coronavirus epidemic in China, Medical support teams backed in 23-Mar ~ 28-Mar, 1-Apr ~ 7-Apr: After 14 days rest

**Table 5:** Multivariate analysis results of depression

Time	Model 1(*cRR*)	Model 2(*aRR*)	Model 3(*aRR*)
10-Feb ~ 20-Feb	Ref	Ref	Ref
3-Mar ~ 5-Mar	0.151(0.072,0.317)*	0.154(0.073,0.325)*	0.152(0.072,0.32)*
15-Mar ~ 18-Mar	0.315(0.219,0.451)	0.32(0.222,0.461)	0.315(0.219,0.454)
23-Mar ~ 28-Mar	0.345(0.277,0.43)	0.34(0.271,0.427)	0.337(0.269,0.423)
1-Apr ~ 7-Apr	0.207(0.164,0.262)***	0.205(0.161,0.261)***	0.204(0.16,0.259)***
*P-trend*	0.701(0.663,0.742)***	0.699(0.659,0.740)***	0.698(0.658,0.739)***

Model 1: Single factor analysis. Model 2: Staff’s sex, age and occupation were adjusted. Model 3: Staff’s sex, age, occupation, marital status, and educational background were adjusted. cRR: cursory Risk Ratio, aRR: adjusted Risk Ratio.

*P < 0.05, **P < 0.01, ***P < 0.0001。

*10-Feb ~ 20-Feb is at the beginning of coronavirus epidemic in China, 3-Mar ~ 5-Mar and 15-Mar ~ 18-Mar is in the period of coronavirus epidemic in China, Medical support teams backed in 23-Mar ~ 28-Mar, 1-Apr ~ 7-Apr: After 14 days rest

## Discussion

### Relationship between time and mental state of medical staff under epidemic situation

According to the survey data shown in Tables 2 and 3, considering the changes of the mental health of medical workers in different stages of the epidemic, it is not difficult to find the following conclusions.

First, compared with other stages, the psychological problems of medical staff are the most common and serious in the initial period. Another study on COVID-19 also proved that the psychological status of surgical staff in the initial period was more severe than that in the latter period (score of depression: 7.333 ± 2.508 vs 4.933 ± 2.154) [13]. SARS [14] and Ebola [15] also showed the same trend of anxiety and depression. The reason for this particular trend might be their concern about their own infection and transmission to their families [16], being equipped with full body protective equipment with negative pressure for more than 12 h, and uncertain and inaccurate news related to COVID-19 [17, 18] at the initial period of the epidemic. According to the experience of SARS in 2003, it is necessary to arrange psychiatrists to provide temporary treatment for medical workers as soon as possible [19].

Second, compared to the previous stage, between the former period of Stages 2 and Stage 3 of the epidemic, psychological problems increased, or the existing problems became more serious. In the same period, the medical staff knew that they were going to leave Hubei Province, be isolated, and face their families [20]. According to previous research, what was the most worrying for medical staff was their families [21]. Being afraid of spreading the virus to family members and 233 friends, high levels of anxiety and depression are unavoidable [18, 22]. Medical workers should be encouraged to accept risks, avoid thinking, relax, and try to maintain a positive attitude [21]. In addition, governments at all levels in China also paid attention to the psychological status of medical staff in this process. Intervention measures include a shift system to reduce workload and providing life and family security support to ease the anxiety [20].

### Characteristics of medical staff who are vulnerable in terms of psychological status under the epidemic situation

The comparative analysis of data showed that some social demographic characteristics, such as, female and unmarried status, were the vulnerable characteristics of anxiety and depression of medical staff. Many studies have confirmed that being female [23, 24] can increase the exposure of medical staff to psychological problems in the COVID-19 epidemic owing to females’ individual perception regarding the seriousness of COVID-19. Unmarried medical staff proved to suffer more loneliness and less social support, while the lonely proved to have more psychological problems [25, 26].

In this study, the psychological problems of medical staff working outside Hubei Province are more serious than those in Hubei Province. Medical staff working outside Hubei Province were mainly investigated in Stage 1, a stage with the highest prevalence of mental problems, while all medical staff working in Hubei Province were investigated in Stages 3 and 4. We still cannot rule out that this conclusion is caused by the mixed influence of the epidemic development period, especially its serious conflict with previous conclusions and common sense. Therefore, following the conclusion of previous studies, we believe that the psychological problems of medical staff working in Wuhan during the epidemic period are more seriou s[27]. The medical staff working in Wuhan are faced with a high risk of infection, overwork, depression, discrimination, weak protection against infection, isolation, exhaustion, lack of contact with their families and patients with negative emotions [20].

Therefore, we suggest that the supervision and intervention of psychological problems for female, young, unmarried medical workers and medical workers working in Wuhan should be priority areas of mental health care for anti-epidemic medical staff, and the psychological supervision and intervention for these people should run through the whole process of mental health care.

### The change trend of psychological status of different groups at a certain stage

The whole population needs attention at some specific stages (Stage 1, the latter period of Stage 2, Stage 3), and some subgroups in individual stages also need specific attention.

In Stage 2 (the development stage of the epidemic), we found that more medical workers with other marital status (divorced, separated, widowed) developed depression, and their depression levels became serious compared with other stages of the same population. A study on workforce returning to work (February 24–25, 2020) also confirmed that people with other marital status (divorced, separated, widowed) had significantly higher anxiety and depression [23]. Obviously, adequate social support provided by a partner or spouse is a protective factor of mental health. In the early stage of the rapid development of the epidemic, social support as a buffer stress response ability is helpful to reduce anxiety and depression for medical workers in the face of epidemic challenges. However, those with other marital status without social support from their spouses lack the ability of facing stress.

In Stages 3 and 4, the trend of problems of medical staff with other education background was the opposite compared with the rest of the study population. Previous findings suggest that there is a correlation between a low educational level and psychological problems [28]. Obviously, our results do not conform to previous perceptions. We believe that the reality should be that during the returning time from Hubei Province, more depression and anxiety will strike people with a low education level. This is because perception with medical expertise may help break COVID-19’s sense of mystery, so as to reduce the panic of infected family members, partners and spouses. Based on this conjecture, we believe that systematic pre-training for the COVID-19 epidemic can help reduce unnecessary psychological problems [24].

## Conclusion

In reviewing the psychological performance of medical personnel in China during the period of COVID-19, it is certain that the psychological problems between the former period of Stage 2 and Stage 3 and in the initial period of the outbreak are serious, which is also the essential period of psychological protection strategy.

In addition, young, unmarried, female medical workers, and medical workers working in Wuhan are more vulnerable in the whole process, needing the attention and additional inclination of psychological medical resources; while the other marital status groups and other educational background groups are particularly vulnerable in a specific stage, which means that the focus of a phased policy is on target.

### Limitations

First of all, the scope of participants is limited, they are all from Yunnan Province, thus the generalization of research results is limited. Secondly, this study is based on online survey and the survey results are self-reported, lack of face-to-face interviews, so there may be missing information; thirdly, this study can not judge whether the psychological problems of participants always exist or appear during the epidemic period; fourthly, the response rate of the research questionnaire is low. The reason may be that the respondents are too stressed or not interested in the research. In this case, response bias may exist. Fifthly, the division of research period and the selection of respondents are limited by the epidemic situation, therefore the interval of each period and the number of respondents are always not consistent.

## Supplementary Information

**Additional file 1. Table S1.** Classification criteria of depression level and anxiety level.

## Data Availability

The datasets generated and/or analysed during the current study are not publicly available due to being applied in the following non-public research but are available from the corresponding author on reasonable request.

## Abbreviations

COVID-2019: Corona Virus Disease 2019
SSS: Somatization Symptom Checklist
PCL-C: Self rating scale for post-traumatic stress disorder
