# Supplementary material for: Dynamic monitor on psychological problems of medical aid teams in the context of corona virus disease 2019: a multi-stage and multi-factor quantitative study
Source: BMC Public Health. 2021 Aug 3;21:1500. doi: 10.1186/s12889-021-11479-0 (PMC8330201; doi:10.1186/s12889-021-11479-0)
Supplement: Supplementary file 1 — Additional file 1. Table S1. Classification criteria of depression level and anxiety level. [file 12889_2021_11479_MOESM1_ESM.doc]

Appendix Table 1. Classification criteria of depression level and anxiety level

| Depression | | | Anxiety | | |
| --- | --- | --- | --- | --- | --- |
| Scale | Scale score | Degree | Scale | Scale score | Degree |
| PHQ-9 | 0-4 | no depression | GAD-7 | 0-4 | no anxiety |
|  | 5-9 | mild depression |  | 5-9 | mild anxiety |
|  | 10-14 | moderate depression |  | 10-13 | moderate anxiety |
|  | 15-19 | severe depression |  | 14-18 | severe anxiety |
|  | >=20 | extreme severe depression |  | >=19 | extreme severe anxiety |
